# The Annual Rhythms in Sleep, Sedentary Behavior, and Physical Activity of Australian Adults: A Prospective Cohort Study

**DOI:** 10.1093/abm/kaae007

**Published:** 2024-02-23

**Authors:** Ty Ferguson, Rachel Curtis, François Fraysse, Timothy Olds, Dorothea Dumuid, Wendy Brown, Adrian Esterman, Carol Maher

**Affiliations:** Alliance for Research in Exercise, Nutrition and Activity (ARENA), University of South Australia, City East Campus, Frome Road, GPO Box 2471, Adelaide, SA, 5001, Australia; Alliance for Research in Exercise, Nutrition and Activity (ARENA), University of South Australia, City East Campus, Frome Road, GPO Box 2471, Adelaide, SA, 5001, Australia; Alliance for Research in Exercise, Nutrition and Activity (ARENA), University of South Australia, City East Campus, Frome Road, GPO Box 2471, Adelaide, SA, 5001, Australia; Alliance for Research in Exercise, Nutrition and Activity (ARENA), University of South Australia, City East Campus, Frome Road, GPO Box 2471, Adelaide, SA, 5001, Australia; Alliance for Research in Exercise, Nutrition and Activity (ARENA), University of South Australia, City East Campus, Frome Road, GPO Box 2471, Adelaide, SA, 5001, Australia; School of Human Movement and Nutrition Sciences, University of Queensland, Brisbane, Queensland 4072, Australia; Alliance for Research in Exercise, Nutrition and Activity (ARENA), University of South Australia, City East Campus, Frome Road, GPO Box 2471, Adelaide, SA, 5001, Australia; Alliance for Research in Exercise, Nutrition and Activity (ARENA), University of South Australia, City East Campus, Frome Road, GPO Box 2471, Adelaide, SA, 5001, Australia

**Keywords:** Sleep, Sedentary behavior, Physical activity, Time-use, Annual rhythms

## Abstract

**Background:**

Sleep, sedentary behavior, and physical activity have fundamental impacts on health and well-being. Little is known about how these behaviors vary across the year.

**Purpose:**

To investigate how movement-related behaviors change across days of the week and seasons, and describe movement patterns across a full year and around specific temporal events.

**Methods:**

This cohort study included 368 adults (mean age = 40.2 years [*SD* = 5.9]) who wore Fitbit activity trackers for 12 months to collect minute-by-minute data on sleep, sedentary behavior, light physical activity (LPA), and moderate-to-vigorous physical activity (MVPA). Data were analyzed descriptively, as well as through multilevel mixed-effects linear regression to explore associations with specific temporal cycles (day-of-the-week, season) and events.

**Results:**

Movement patterns varied significantly by day-of-the-week and season, as well as during annual events like Christmas-New Year and daylight saving time (DST) transitions. For example, sleep was longer on weekends (+32 min/day), during autumn and winter relative to summer (+4 and +11 min/day), and over Christmas-New Year (+24 min/day). Sedentary behavior was longer on weekdays, during winter, after Christmas-New Year, and after DST ended (+45, +7, +12, and +8 min/day, respectively). LPA was shorter in autumn, winter, and during and after Christmas-New Year (−6, −15, −17, and −31 min/day, respectively). Finally, there was less MVPA on weekdays and during winter (−5 min/day and −2 min/day, respectively).

**Conclusions:**

Across the year, there were notable variations in movement behaviors. Identifying high-risk periods for unfavorable behavior changes may inform time-targeted interventions and health messaging.

## Teaser

Adults’ sleep, sedentary behaviour and physical activity fluctuates significantly across weekly, seasonal and annual cycles, and around events such as the Christmas to New Years period

## Introduction

Time use during a 24-h day can be divided into three broad categories: sleeping, being sedentary, and being active [1]. The amount of time spent in each influences health [2]. International guidelines suggest adults should engage in the equivalent of 150–300 min of moderate physical activity or 75–150 min of vigorous physical activity per week, 7–9 h of sleep per night, and limit prolonged bouts of sedentary behavior [3–5]. Emerging evidence also supports the benefits of light physical activity (LPA) [6, 7]. Conversely, insufficient physical activity or sleep, and excessive sleep or sedentary time are associated with negative health outcomes, including increased mortality and morbidity, increased incidence of non-communicable chronic disease, and poor mental health [6, 8–10]. Interventions are needed to improve movement patterns, and these interventions should acknowledge and address the factors which influence and constrain movement behaviors.

Allocation of time to each movement behavior reflects a complex and dynamic interaction of internal and external factors. These include the physical environment (e.g., urban design, natural environment, and weather) [11], resource availability (e.g., finances, exercise or active transport equipment, home design), time constraints (e.g., employment, care duties, unpaid work, unplanned disruptions), and physical (e.g., fitness, health status, presence of injury, or disability) [12], and cognitive attributes (e.g., values, beliefs, personality, motivators, and barriers). As a result, the day-to-day distribution of time is susceptible to fluctuations. Literature on intra-annual patterns is sparse.

Our recent systematic review identified 17 studies of intra-annual temporal cycles, specifically seasonal changes, and temporal events, including Ramadan, vacations, and daylight saving time (DST) transitions [13]. Several key limitations with the existing evidence base were identified. Namely, a small range of temporal factors was explored in isolation, most studies did not report the full range of daily movement behaviors (94%), and generally, only short measurement periods were used (82% measured 2 weeks or less at each timepoint). These limitations reduce the clarity and strength of findings and increase the potential for external effects on behavior (e.g., unseasonable weather, brief unexpected life events).

Gaining an understanding of the fluctuations of movement patterns within a year-long cycle is important for informing the timing of interventions and health promotion initiatives. Delivering interventions and health messaging at the time they are most needed will have the greatest benefit. Further, identifying temporal factors associated with unfavorable movement behaviors may afford additional opportunities for positive health action.

Our aim was to explore how daily movement patterns of Australian adults vary across days of the week and seasons, over a full year, and around specific society-wide temporal events. Building on the temporal events highlighted in our previous systematic review [13], we honed in on the Christmas to New Year’s Day period, a distinctive 7-day period which includes 3-weekday public holidays, DST transitions, and school holidays. This study is exploratory, and whilst the current evidence base provides clues for expected associations, (i.e., sleep and sedentary behavior increase while physical activity decreases in winter) no studies have explored the effects of temporal events and cycles on movement patterns in a southern hemisphere population.

## Methods

### Study Design

Data used in this study were collected for the *Annual Rhythms In Adults’ lifestyle and health* (ARIA) prospective cohort study. ARIA followed a cohort of Australian adults, collecting data on 24-h movement behaviors, dietary patterns, body weight, and well-being. Further details of the study protocol have been published elsewhere [14].

Ethics approval was provided by the University of South Australia Human Research Ethics Committee (Protocol number: 201901). All participants provided informed, written consent.

### Setting and Participants

The ARIA study recruited 375 community-based, middle-aged adults from Adelaide, South Australia, either as parents of children enrolled in a separate cohort study called Life on Holidays [15] (cohorts 1 and 2), or parents of primary school children recruited from the community via general advertising (cohort 3). The study enrolled participants in two waves, with cohort 1 commencing on December 1, 2019, and cohorts 2 and 3 commencing on December 1, 2020. Waves remained in the study for 13 months, finishing on December 31 of the year after enrollment.

Inclusion criteria required participants to be aged 18–65, a parent/guardian of a child enrolled in the Life on Holidays study or a parent/guardian of a child aged 5–12 years, residing in greater metropolitan Adelaide, have access to a Bluetooth-enabled mobile phone or computer and home internet, understand the English language, and be ambulant. Exclusion criteria included pregnancy, having an implanted electronic medical device, or receiving treatment for, or experiencing any life-threatening condition which impacted daily lifestyle and health.

Participants in the study were visited at their homes where they provided baseline information about their health and lifestyle and measured for height. Participants were provided with a Fitbit Charge 3 fitness tracker and Fitbit Aria body weight scale (Aria 2 or Aria Air scale, Fitbit Inc., San Francisco, CA, USA) and instructed in their daily use.

### Variables

#### Daily 24-h movement behavior

Minute-level movement behavior data were collected via a Fitbit Charge 3 activity tracker worn on the non-dominant wrist. Fitbit data were collected remotely via purpose-built “Fitnesslink” software.

Fitbit’s proprietary algorithm pre-classified every minute to one of five intensity-based behaviors; including sleep, sedentary behavior, LPA, moderate physical activity, and vigorous physical activity. Fitbit’s proprietary algorithm is not publicly available. Daily summaries for each behavior were calculated. Minutes of sedentary behavior without heart rate data were reclassified as non-wear. To account for minutes of non-wear, each day was normalized by averaging all behaviors up to a total of 1,440 minutes. Participants were retained in the data set if they had at least 2 valid days during the study period, to ensure collection of repeated measures, where a valid day has at least 18 h of movement data and a period of sleep. This threshold was strategically chosen to ensure a balance between precision and maximization of participant retention. Fitbit devices have shown acceptable validity for sleep duration [16, 17], sedentary behavior [18], and physical activity [19, 20].

#### Temporal cycles and events

Temporal cycles explored statistically included day-of-the-week, season, and school holidays. Temporal events included DST transitions and the Christmas-New Year period.

#### Participant characteristics

Participant baseline data included age, sex, chronic conditions, smoking status, indigenous status, country of birth, marital status, number of adults, and children in the household, highest education level, occupation, hours of work per week outside the home, and combined gross household income.

### Statistical Analysis

Multilevel mixed-effects linear regression modeling with random intercepts was used to compare movement behaviors across days of the week, seasons, and “events,” with movement behaviors as the dependent variables. This statistical approach was chosen to account for the non-independence of the data, account for nesting of measures within individuals, families, and study waves, and included both fixed and random effects. Day-of-the-week, season, and “events” (holiday period and daylight saving time), were included as fixed effects. Days of the week analyses compared Monday with Tuesday through to Sunday, with Monday being considered the commencement of the typical working week. Season compared mid-summer (January) with the middle month of each remaining season (autumn = April, winter = July, spring = October). Christmas-New Year periods compared the 7 days prior to Christmas (December 18–24) to the Christmas-New Year period (25th Dec–1st Jan) and the 7 followings days (January 2–8). Daylight saving time analyses compared the 7 days prior to each transition day with the 7 following days (including the day of transition and the next 6 days). School holidays analyses compared all holiday periods, summer and mid-term combined, to all school term periods [21]. Multilevel models used all available data for the chosen time period for each analysis.

Annual rhythms in each movement behavior were examined as a change from the yearly mean, using a 14-day moving average.

Statistical modeling was completed in Stata 17 (StataCorp, College Station, TX, USA) with statistical significance set at 0.05. Graphs were generated using MATLAB version 9.11 (MathWorks, Natick, MA, USA).

## Results

### Participants

Movement behavior data were available for 368 participants (Table 1) with an average age was 40 years. The majority were overweight or obese (34% and 35%, respectively), married or in de facto relationships (85%), and had two or more children (90%). Almost half the participants had a university education (48%).

**Table 1:** Participant Characteristics at Baseline (*n* = 368)

	Mean (*SD*)
Age (years)	40.2 (5.9)
Weight (kg)	84.0 (20.5)
Height (cm)	170.4 (9.5)

	*n* (%)
Sex	
Female	209 (56.8)
Male	159 (43.2)
Weight status	
Underweight	1 (0.3)
Normal	114 (31.0)
Overweight	124 (33.7)
Obese	129 (35.1)
Chronic illness	
None	163 (44.3)
Single	107 (29.1)
Multiple	98 (26.6)
Sleep chronotype	
Definitely a morning type	82 (22.3)
More a morning type than evening type	122 (33.2)
More an evening type than morning type	111 (30.2)
Definitely an evening type	53 (14.4)
Smoker	34 (9.2)
Aboriginal and Torres Strait Islander peoples	4 (1.1)
Born in Australia	279 (75.8)
Marital status	
Married/de facto	313 (85.1)
Separated, divorced or widowed	29 (7.8)
Never married	26 (7.1)
Adults in household	
One	38 (10.3)
Two	305 (82.9)
Three	17 (4.6)
Four or more	7 (1.9)
Children in household	
One	36 (9.8)
Two	195 (53.0)
Three	95 (25.8)
Four or more	42 (11.5)
Education	
Year 10 or less	17 (4.6)
Year 11–12	49 (13.3)
Certificate/Diploma	125 (34.0)
University degree	177 (48.1)
Occupation	
Managerial and professional	148 (40.2)
Technical and clerical	77 (20.9)
Community, personal service, sales	67 (18.2)
Machinery operators, drivers, laborer	14 (3.8)
No job/other	62 (16.8)
Hours worked per week	
None	55 (14.9)
<15	25 (6.8)
15–35	119 (32.3)
36+	169 (45.9)
Household income (AUD)	
< $50,000	37 (10.1)
Between $50,000 and $99,999	111 (30.2)
Between $100,000 and $199,999	176 (47.8)
≥$200,000	44 (12.0)

### Movement Behavior Data

Data from 92,912 valid days were analyzed, with an average of 252 (*SD* = 116, median = 288, IQR = 165–349, range = 2–395) days per participant (see Supplementary Fig. S1). The average percentage of valid daily participant data was 63.7% (*SD* = 6.6%), declining from an average of 73% in the first month to an average of 51% in the thirteenth month. A detailed daily breakdown is included as Supplementary Fig. S2. The daily averages for each movement behavior were 8 h and 10 min of sleep (*SD* = 88 min), 10 h and 18 min of sedentary behavior (*SD* = 121min), 5 h of LPA (*SD* = 92 min) and 32 min of moderate-to-vigorous physical activity (MVPA) (*SD* = 38 min).

### Day-of-the-week

**Fig. 1.**
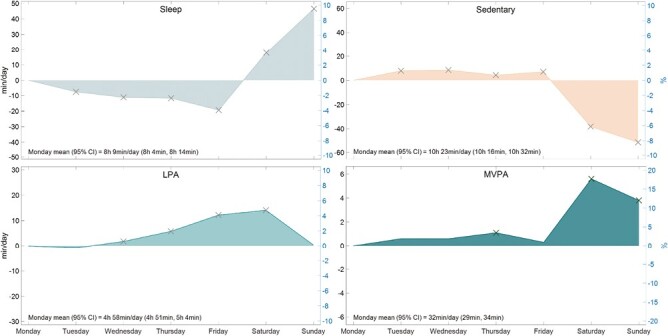
Day-of-the-week modeled changes, compared with Monday (*n* = 368). Total days included in analysis = 92,912. Mean (range) of days contributed per participant = 252 days (2–395 days). *LPA* light physical activity; *MVPA* moderate-to-vigorous physical activity. *X* = significant change from Monday (*p* < .05). Percent range (right *y*-axis ± 10% except for MVPA which is ± 20%).

Daily sleep (midnight-to-midnight) varied by approximately 66 min/day (13.5%) across the week, with the shortest sleep on Fridays (−19 min/day [−3.9%], *p* < .001) and longest on Sundays (+47 min/day [+9.5%], *p* < .001) relative to Mondays (see Fig. 1). There was a clear pattern for sleep to gradually decrease from Monday to Friday, followed by a sharp increase on weekends.

Sedentary behavior varied by approximately 60 min/day (9.6%) across the week, with the least on Sundays (−51 min/day [or −8.2%], *p* < .001) and most on Wednesdays (+8 min/day [+1.4%], *p* < .001) relative to Mondays.

Light physical activity varied by approximately 14 min/day (4.7%) across the week, with a steady upward trajectory from Wednesday to Saturday (+2 min/day [+0.6%] on Wednesday to +14 min/day [+4.7%] on Saturday).

Moderate-to-vigorous physical activity varied by approximately 6 min/day (18%) across the week, with Thursday, Saturday, and Sunday each had significantly more MVPA relative to Monday (+1 min/day, +6 min/day, and +4 min/day, respectively, all *p* < .01).

### Season

**Fig. 2.**
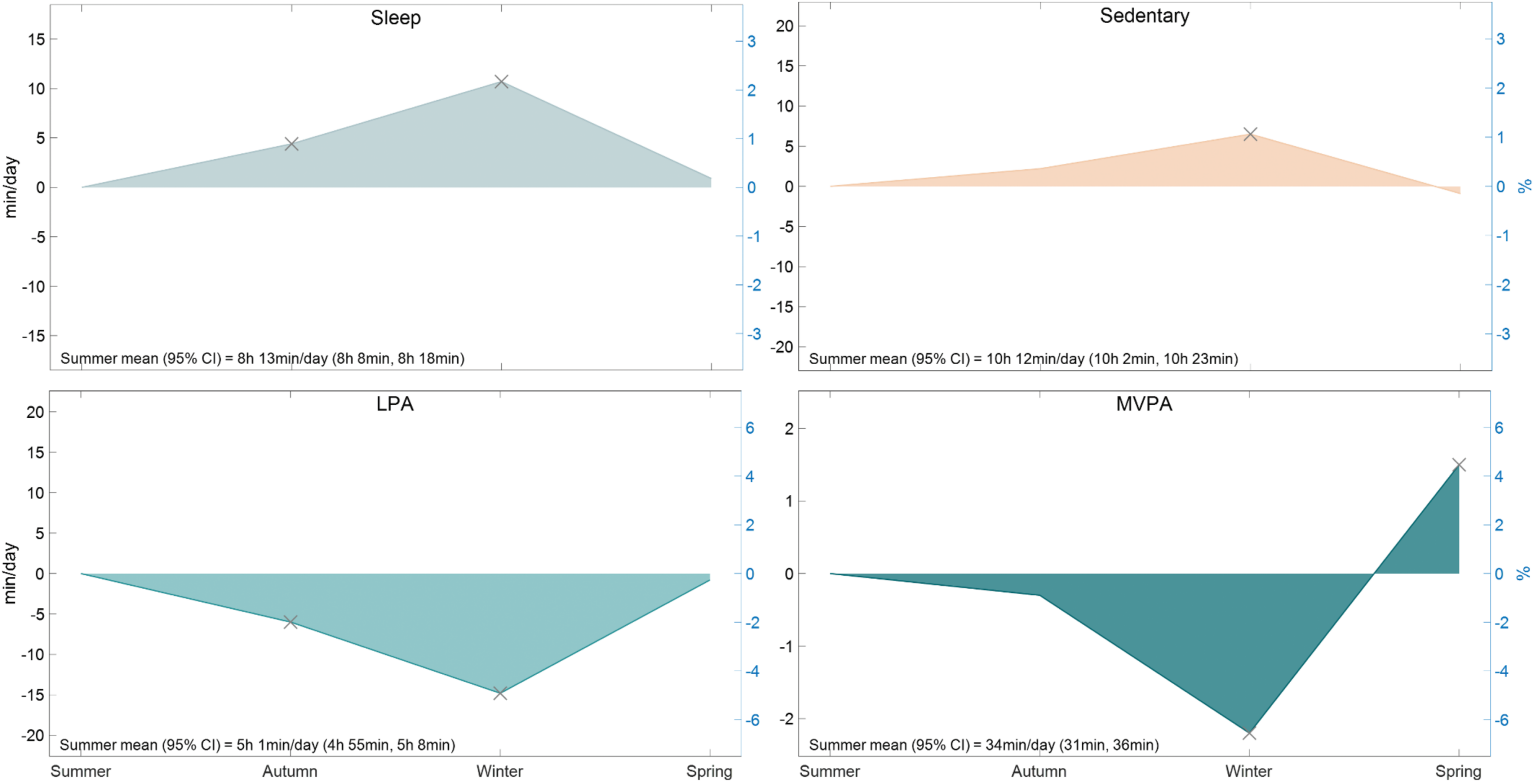
Seasonal modeled changes as compared to summer (*n* = 363). Total days included in analysis = 29,300. Mean (range) of days contributed per participant = 81 days (1–123 days). *LPA* light physical activity; *MVPA* moderate-to-vigorous physical activity. *X* = change from summer (*p* < .05). Percent range (right *y*-axis) is ± 3% for sleep and sedentary, ± 6% for LPA and MVPA.

Sleep was longer in autumn (+4 min/day, +0.9%, *p* = .001), and winter (+11 min/day, +2.2%, *p* < .001), relative to summer (Fig. 2). Sedentary behavior was greater in winter than in summer (+7 min/day, +1.1%, *p* < .001). Light physical activity was less in autumn (−6 min/day, −2%, *p* < .001) and winter (−15 min/day, −4.9%, *p* < .001), than in summer. Moderate-to-vigorous physical activity was less in winter (−2 min/day, −6.5%, *p* < .001), and higher in spring (+2 min/day, +4.5%, *p* = .008) compared with summer.

### Annual Change in 24-h Movement Behaviors

**Fig. 3.**
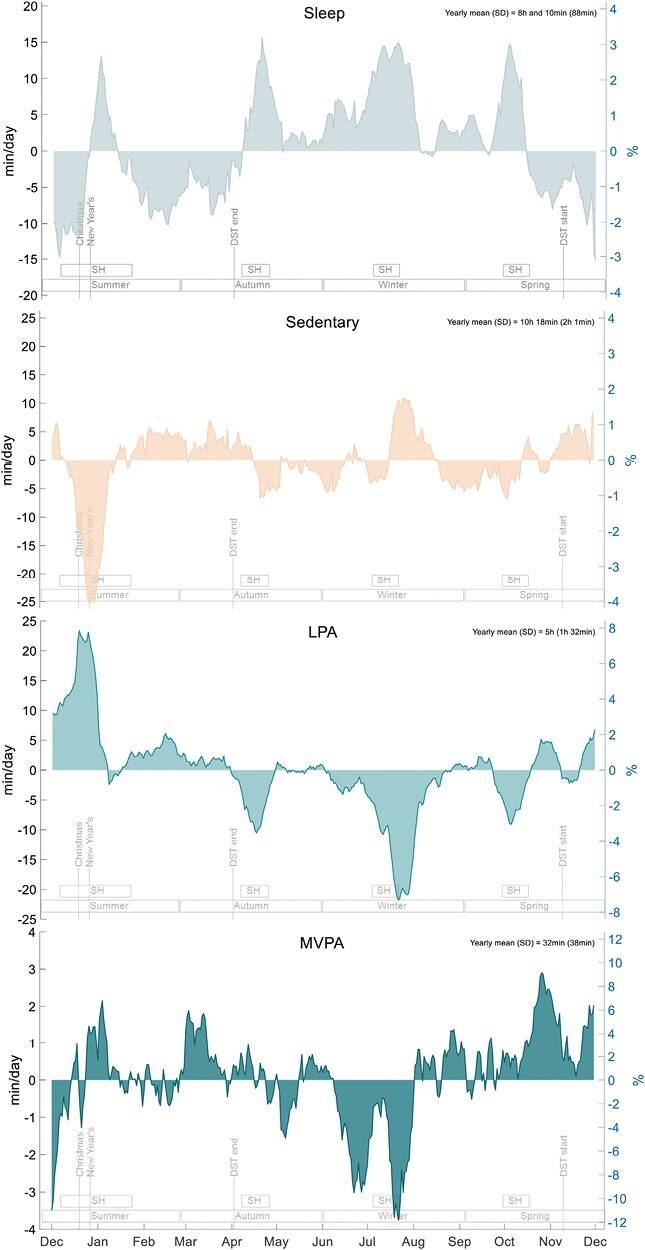
Annual rhythms of sleep, sedentary behavior, LPA, and MVPA (*n* = 368). Presented as change from yearly mean in minutes (left *y*-axis) and as a percent (right *y*-axis) using a centered 14-day moving average. *DST end* daylight saving time ends (clocks moved back 1 h); *DST start* daylight saving time starts (clocks moved forward 1 h); *SH* school holidays.

Annual patterns in 24-h movement behaviors are presented in Fig. 3 and a stacked plot combining all behaviors is included as Supplementary Fig. S3. Sleep varied by approximately 30 min/day (6% of mean sleep duration), with higher values from April to October, and lower values from November to March.

Sedentary behavior varied by approximately 35 min/day (6%) across the year. There were two clear spikes in sedentary behavior, a 1-week dip of approximately −25 min/day (−4%) around Christmas to New Year, and a 1-week spike of approximately 10 min/day (+1.8%) in late July.

Light physical activity varied by approximately 43 min/day (14%) across the year. Light physical activity was generally above average from November to March, and below average from April to October, with a spike around Christmas to New Year (+23 min/day, +7.7%) and a dip in late July (−20 min/day, −6.7%).

Moderate-to-vigorous physical activity varied by approximately 7 min/day (21%) across the year and appeared slightly lower in June and July and higher from August to November. A spike occurred at the end of October (+3 min/day, +9%), and dips occurred in July and August (approximately, −3 min/day, −9% and −4 min/day, −12%, respectively).

### Christmas-New Year Holiday Period

During the Christmas-New Year holiday period, sleep increased by 24 min per day (+5.0%, *p* < .001), while LPA decreased by 17 min/day (−5.2%, *p* < .001), compared to the week before. After the holiday period, sleep increased by 21 min/day (+4.3%, *p* < .001), LPA decreased by 31 min/day (+9.4%, *p* < .001), and sedentary behavior increased by 12 min/day (+2%, *p* < .001) compared to the week before the holiday period (see Table 2).

**Table 2:** Temporal Events Comparisons

	Coefficient	[95% conf. interval]	*p*
Christmas-New Year *n* = 353, total days = 6,080, mean (range) per participant = 17 days (1–35 days)			
Sleep			
Pre (cons)	479.2	[473.0, 485.4]	
During	**23.8**	[19.0, 28.6]	<.001
Post	**20.7**	[15.7, 25.7]	<.001
Sedentary			
Pre (cons)	597.3	[588.7, 605.9]	
During	−4.5	[−10.3, 1.2]	.121
Post	**12.0**	[5.9, 18.0]	<.001
LPA			
Pre (cons)	331.2	[321.3, 341.1]	
During	**−17.2**	[−21.5, −12.9]	<.001
Post	**−31.1**	[−35.6, −26.5]	<.001
MVPA			
Pre (cons)	35.5	[32.7, 38.2]	
During	−1.8	[−3.8, 0.2]	.079
Post	−1.6	[−3.7, 0.5]	.130

	Coefficient	[95% conf. interval]	*p*
DST start *n* = 291, total days = 3,006, mean (range) per participant = 10 days (1–14 days)			
Sleep			
Pre (cons)	504.8	[497.4, 512.2]	
Post	4.4	[−1.3, 10.1]	.129
Sedentary			
Pre (cons)	606.3	[586.2, 626.4]	
Post	−4.3	[−11.6, 3.0]	.244
LPA			
Pre (cons)	292.6	[284.1, 301.0]	
Post	−1.5	[−6.6, 3.5]	.552
MVPA			
Pre (cons)	33.7	[30.7, 36.8]	
Post	−1.0	[−3.4, 1.4]	.416
DST end *n* = 333, total days = 3,434, mean (range) per participant = 10 days (1–14 days)			
Sleep			
Pre (cons)	490.5	[484.1, 496.8]	
Post	**−7.2**	[−12.6, −1.8]	.009
Sedentary			
Pre (cons)	619.0	[609.1, 628.9]	
Post	**7.6**	[1.0, 14.2]	.024
LPA			
Pre (cons)	299.2	[291.1, 307.3]	
Post	−0.5	[−5.0, 4.1]	.839
MVPA			
Pre (cons)	32.9	[30.1, 35.7]	
Post	−0.2	[−2.4, 2.0]	.857

Results of multilevel mixed-effects linear regression analyses (one model per movement behavior, per comparison). Models adjusted for nesting of observations within participants, within families, within study waves. Bold values indicate *p* < .05. *Christmas-New Year*: pre = 7 days prior to December 25, during = December 25 to January 1, post = 7 days post January 1. *Daylight saving time (DST) end*: clocks shifted backward 1 hour on April 5, 2020 and April 4, 2021, pre = 7 days prior to transition, post = 7 days post. *DST start*: clocks shifted backward 1 hour on October 4, 2020 and October 3, 2021, pre = 7 days prior, post = 7 days post.

*Cons* constant; *LPA* light physical activity; *MVPA* moderate-to-vigorous physical activity.

### Daylight Saving Transitions

The weeks before and after the start of daylight saving time, when clocks shifted 1 h forward, did not differ significantly for any movement behavior. At the end of daylight saving time, when clocks shifted 1 h backward, sleep was shorter in the week after by 7 min/day (−1.5%, *p* = .009) and sedentary behavior was 8 min/day longer (+1.2%, *p* = .024), relative to the week before the transition (see Table 2).

### School Holidays

During school holidays, sleep was 11 minutes a day longer (+2.3%, *p* < .001), sedentary behavior was 12 minutes a day shorter (−2.0%, *p* < .001), and MVPA was less than a minute per day longer (+0.5 min/day, +1.5%, *p* = .043) than non-school holiday periods. Light physical activity did not significantly differ from non-school holiday periods (see Table 2).

### Gender

When considering daily movement behaviors by gender, women averaged significantly more sleep (8 h 24 min/day vs. 7 h 53 min/day, *p* < .001) and LPA (5 h 13 min/day vs. 4 h 52 min/day, *p* = .002), and less sedentary behavior (10 h 1 min/day vs. 10 h 29 min/day, *p* < .001), and MVPA (23 min/day vs. 47 min/day, *p* < .001) per day than men across the study period. Temporal analyses showed direction and magnitude of change were generally consistent between genders, with several exceptions. Light physical activity peaked on Friday for women versus Saturday for men. Moderate-to-vigorous physical activity on weekends was 8 to 9 min/day longer for men than women. Sedentary behavior was shorter for women and longer for men during autumn, winter, and spring, relative to summer. Light physical activity was significantly shorter for men across all seasons relative to summer (−23 to -36 min/day, all *p* < .001), with only winter differing significantly for women (−10 min/day, *p* = .001). Women were significantly more sedentary after the Christmas to New Year’s period (+17 min/day, *p* < .001), with men significantly less sedentary during the period (−11 min/day, *p* = .016), relative to pre-Christmas. Light physical activity was significantly shorter for females during the Christmas to New Year’s period, relative to pre-Christmas (−28 min/day, *p* < .001) (see Supplementary Table S3).

## Discussion

In this cohort study, we examined 12 months of movement behavior data to understand day-to-day and seasonal changes and explore changes related to temporal events such as Christmas-New Year and daylight savings time transition periods. Movement behaviors showed clear fluctuations during these events, as well as a weekly and seasonal rhythm. Notably, sleep was longer on weekends, during autumn and winter, and over Christmas-New Year, while sedentary behavior was longer on weekdays, during winter, and after Christmas-New Year and DST ended. Light physical activity was shorter in autumn, winter, and during and after Christmas-New Year, and MVPA was shorter on weekends and during winter.

The weekday–weekend and seasonal rhythms in movement behaviors observed in this study are broadly consistent with previous literature. For example, longer sleep on weekends has been reported in previous studies [22–24]. A systematic review by Chaput et al. [25] provided evidence of catch-up sleep as favorable for health, however, Putilov et al. [22] concluded that it is not possible to fully recover sleep debt on weekends. A pattern of more active and less sedentary weekends [26] is sometimes described as a “weekend warrior” profile [27], which is associated with increased leisure time on non-work days [28].

In contrast to our previous systematic review and meta-analysis which reported non-significant seasonal changes in 15 out of 16 comparisons (*p* ≥ .05) [13], our current study identified significant seasonal changes in all four daily movement behaviors, amounting to up to 15 min/day. It is possible that our larger sample size or the use of summer as a comparator (instead of the yearly average) might account for the observed differences in significance. A recent scoping review of seasonal variations in physical activity and sedentary behavior reported broadly similar results to the current study, with moderate to strong evidence in support of our findings [29]. More sleep and sedentary behavior and less physical activity during winter than in summer is likely a result of less favorable weather conditions for outdoor leisure, exercise, and active transport [11, 30]. Additionally, extremes of weather (i.e., too hot, cold, wet, or windy) are more likely to push people indoors where sedentary activities are more commonly performed [31].

The societal events explored in this study identified specific timepoints of movement pattern fluctuations. The week spanning Christmas to New Year’s Day is a socially significant period of change in time demands in Australia. It is marked by 3-weekday public holidays within a 7-day span [32], heightened vacation and travel (61% of our study’s participants took at least one vacation day during this period) [33], and a slowdown in non-essential industries [34]. Our study appears to be the first to explore movement behavior duration changes at this period. However, a 2000 study of US adults’ holiday weight gain (including Thanksgiving) reported a decrease in physical activity levels during holidays [35], comparable to the decrease in LPA observed in our study.

Previous research on DST transitions is limited, with only one study identified. Tonetti et al. [36] reported a much larger increase in sleep (+25 min/day) post-DST ending than this study (+7 min/day), but their study included a small sample of young male university students (*n* = 14) who had an average sleep duration of 2 h/day less than the ARIA participants who were both males and females. The time demands and time-use behaviors of the Tonetti sample are likely to be very different from those of the middle-aged, generally employed parents in the ARIA sample. Beyond sleep duration, there is evidence to suggest the start of DST negatively impacts sleep quality along with indirect evidence of increases in traffic accidents attributed to fatigue alertness, ambient light changes, and/or rushing to work [37, 38].

Gender-specific results in our study are largely consistent with existing literature on gender differences in movement behavior patterns. Typically, women are less active than men [39], with more of their physical activity minutes spent on childrearing and domestic duties than leisure activities, which is the opposite for men [40]. Notably, women in our study averaged less than half the MVPA of men. with men more likely to have a weekend warrior approach to physical activity, again this is consistent with previous literature [28]. Additionally, the lower levels of MVPA in women may be due to the division of parenting duties with previous literature suggesting women who have children at home are less active than those without [41], a difference not observed in men [40].

### Strengths and Limitations

The main strength of this study is the collection of continuous minute-by-minute device-measured movement behavior data over a whole year. These continuous data afford the ability to assess changes in movement behaviors due to various temporal factors at a granular level. Limitations include that data collection occurred during the COVID-19 pandemic, where changes to work patterns and access to usual active pursuits (e.g., gyms, sports leagues) were limited at times. Though, as Australia maintained an “elimination” approach to the virus (i.e., closed international and state borders, strict quarantine rules for new arrivals) for much of the study period, there were minimal lifestyle and schooling impacts in South Australia, compared with other states and countries. When considering generalizability, it’s important to acknowledged that participants were all approximately middle-aged parents of children age 5–12 residing in one major Australian city located in a temperate climate zone. Therefore, findings may not be representative of adults without children (approximately, 23% of middle-aged Australians), those residing in non-urban areas (14% of Australians), and in non-temperate climates.

### Implications and Future Directions

From a theoretical standpoint, our study suggests that time-use behaviors tend to remain constant unless disrupted by events or changes in temporal cycles. Environmental and social factors can play a significant role in altering these movement behaviors. This observation aligns with theories emphasizing the importance of contextual cues in driving behavior change. As demonstrated by Kyung et al. [42], real-time weather data can be a powerful tool to tailor mobile health interventions to enhance physical activity.

From a scientific perspective, our study pioneers a fresh methodological approach. Advances in wearable technology have made it possible to delve deep into temporal patterns of daily movement behaviors across a year in a large cohort. This innovation allows us to understand these patterns with unprecedented accuracy, shedding light on the context and potential health risks associated with different movement behaviors. In doing so, we uncovered findings which suggest that future research should aim to encompass full temporal cycles when possible (e.g., record at least 7 days) and adjust for seasonal variations in their analyses.

From a practical and policy perspective, our findings offer valuable guidance for crafting effective public health strategies. Harnessing real-time data, such as weather information, to customize public health messages might amplify their impact. Policymakers could strategically focus on periods like winter and the Christmas-New Year stretch to promote healthier movement behaviors. Specifically, interventions might emphasize the importance of staying active during winter, maintaining consistent movement patterns on weekdays, ensuring adequate sleep, especially during DST transitions, and adopting healthier behaviors around the festive season.

Future research should explore additional temporal events and climates’ impact on daily movement patterns with larger and more diverse samples. Examining intra-day variability in movement patterns and its relationship with health outcomes, such as mental health, would also be valuable.

Finally, the Christmas-New Year period appears to be an important time for short-term movement behavior changes. It is however a time when many people take vacations. Studying the association between vacations and movement behaviors may highlight a potentially important context for intervention development. Building on this, it would be valuable for future research to examine if certain periods, such as traditional “start times” like Mondays or New Year, serve as more effective intervention points, given their psychological significance as fresh beginnings for individuals. This could offer more strategic, tailored intervention designs based on behavioral patterns during specific times of the year.

## Conclusion

This study shows that changes in sleep, sedentary behavior, and physical activity throughout the year are linked to events like Christmas-New Year and daylight saving time transitions, in addition to weekly and seasonal rhythms. With precise monitoring of daily movement patterns, we identified specific periods of highest risk for unfavorable behavior changes. This knowledge has the potential to inform the timing of interventions and public health messaging to ensure that supports for optimal movement patterns are delivered at the time they are most needed.

## Supplementary Material

kaae007_suppl_Supplementary_Material

## Transparency statements

(1) This secondary analysis study was not formally registered. The larger study from which data was derived was pre-registered on the Australian New Zealand Clinical Trial Registry (Trial ID: ACTRN12619001430123). https://www.anzctr.org.au/Trial/Registration/TrialReview.aspx?id=378060&isReview=true. (2) The analysis plan was not formally pre-registered. (3) De-identified data from this study are not available in an a public archive. De-identified data from this study will be made available (as allowable according to institutional IRB standards) by emailing the corresponding author. (4) Analytic code used to conduct the analyses presented in this study are not available in a public archive. They may be available by emailing the corresponding author. (5) Materials used to conduct the study are not publically available.
