# Supplementary material for: The Annual Rhythms in Sleep, Sedentary Behavior, and Physical Activity of Australian Adults: A Prospective Cohort Study
Source: Ann Behav Med. 2024 Feb 23;58(4):286–95. doi: 10.1093/abm/kaae007 (PMC10928835; doi:10.1093/abm/kaae007)
Supplement: kaae007_suppl_Supplementary_Material [file kaae007_suppl_supplementary_material.docx]

**Supplementary files**

**Table S1: Day of the week comparisons**

| **Day of the week** | | | | |
| --- | --- | --- | --- | --- |
|  |  | Coefficient | [95% conf. interval] | p |
| **Sleep** | Monday (cons) | 489.4 | [484.4, 494.4] |  |
|  | Tuesday | **-7.5** | [-9.3, -5.7] | <0.001 |
|  | Wednesday | **-11.1** | [-12.9, -9.3] | <0.001 |
|  | Thursday | **-11.6** | [-13.4, -9.8] | <0.001 |
|  | Friday | **-19.3** | [-21.1, -17.5] | <0.001 |
|  | Saturday | **17.9** | [16.1, 19.8] | <0.001 |
|  | Sunday | **46.6** | [44.8, 48.4] | <0.001 |
| **Sedentary** | Monday (cons) | 623.9 | [616.2, 631.6] |  |
|  | Tuesday | **7.9** | [5.6, 10.1] | <0.001 |
|  | Wednesday | **8.5** | [6.2, 10.7] | <0.001 |
|  | Thursday | **4.3** | [2.0, 6.5] | <0.001 |
|  | Friday | **7** | [4.7, 9.3] | <0.001 |
|  | Saturday | **-38.2** | [-40.5, -35.9] | <0.001 |
|  | Sunday | **-51.2** | [-53.5, -48.9] | <0.001 |
| **LPA** | Monday (cons) | 297.6 | [291.0, 304.2] |  |
|  | Tuesday | -0.8 | [-2.4, 0.9] | 0.356 |
|  | Wednesday | **1.8** | [0.1, 3.4] | 0.037 |
|  | Thursday | **5.8** | [4.2, 7.5] | <0.001 |
|  | Friday | **12.2** | [10.6, 13.9] | <0.001 |
|  | Saturday | **14.1** | [12.4, 15.8] | <0.001 |
|  | Sunday | 0.4 | [-1.3, 2.1] | 0.663 |
| **MVPA** | Monday (cons) | 31.6 | [29.4, 33.9] |  |
|  | Tuesday | 0.6 | [-0.1, 1.4] | 0.113 |
|  | Wednesday | 0.6 | [-0.1, 1.4] | 0.105 |
|  | Thursday | **1.1** | [0.3, 1.9] | 0.006 |
|  | Friday | 0.3 | [-0.5, 1.1] | 0.483 |
|  | Saturday | **5.6** | [4.8, 6.4] | <0.001 |
|  | Sunday | **3.8** | [3.0, 4.6] | <0.001 |

Notes: Bold values indicate p<0.05. Cons = constant, LPA = light physical activity, MVPA = moderate-to-vigorous physical activity.

**Table S2: Seasonal comparisons**

| **Season** | | | | |
| --- | --- | --- | --- | --- |
|  |  | Coefficient | [95% conf. interval] | p |
| **Sleep** | Summer (cons) | 492.8 | [487.7, 498.0] |  |
|  | Autumn | **4.4** | [1.8, 7.0] | 0.001 |
|  | Winter | **10.7** | [8.1, 13.3] | <0.001 |
|  | Spring | 0.9 | [-1.8, 3.5] | 0.518 |
| **Sedentary** | Summer (cons) | 612.2 | [601.5, 622.9] |  |
|  | Autumn | 2.2 | [-1.0, 5.4] | 0.178 |
|  | Winter | **6.5** | [3.3, 9.8] | <0.001 |
|  | Spring | -0.9 | [-4.2, 2.4] | 0.581 |
| **LPA** | Summer (cons) | 301.2 | [294.5, 307.8] |  |
|  | Autumn | **-6** | [-8.2, -3.7] | <0.001 |
|  | Winter | **-14.8** | [-17.1, -12.5] | <0.001 |
|  | Spring | -0.8 | [-3.1, -1.6] | 0.52 |
| **MVPA** | Summer (cons) | 33.6 | [31.2, 35.9] |  |
|  | Autumn | -0.3 | [-1.3, 0.8] | 0.615 |
|  | Winter | **-2.2** | [-3.3, -1.2] | <0.001 |
|  | Spring | **1.5** | [0.4, 2.5] | 0.008 |

Notes: Bold values indicate p<0.05. Cons = constant, LPA = light physical activity, MVPA = moderate-to-vigorous physical activity.

**Table S3: Gender comparisons**

| **Day of the week** | | | | | | | |
| --- | --- | --- | --- | --- | --- | --- | --- |
|  |  | **Female (n = 209)** | | | **Male (n = 159)** | | |
|  |  | Coeff | [95% CI] | p | Coeff | [95% CI] | p |
| **Sleep** | Monday (cons) | 503.6 | [497.7, 509.4] | <0.001 | 469.5 | [462.6, 476.4] | <0.001 |
|  | Tuesday | **-8.1** | [-10.5, -5.8] | <0.001 | **-6.6** | [-9.3, -3.8] | <0.001 |
|  | Wednesday | **-11.6** | [-13.9, -9.2] | <0.001 | **-10.4** | [-13.2, -7.7] | <0.001 |
|  | Thursday | **-12.0** | [-14.4, -9.6] | <0.001 | **-10.9** | [-13.7, -8.2] | <0.001 |
|  | Friday | **-18.9** | [-21.3, -16.5] | <0.001 | **-19.7** | [-22.5, -17] | <0.001 |
|  | Saturday | **15.7** | [13.4, 18.1] | <0.001 | **21.2** | [18.3, 24] | <0.001 |
|  | Sunday | **44.8** | [42.4, 47.2] | <0.001 | **49.2** | [46.3, 52] | <0.001 |
| **Sedentary** | Monday (cons) | 608.7 | [598.9, 618.4] | <0.001 | 644.3 | [632.5, 656.1] | <0.001 |
|  | Tuesday | **8.7** | [5.7, 11.6] | <0.001 | **6.7** | [3.1, 10.2] | <0.001 |
|  | Wednesday | **8.4** | [5.4, 11.3] | <0.001 | **8.6** | [5.1, 12.2] | <0.001 |
|  | Thursday | 2.9 | [0, 5.9] | 0.052 | **6.1** | [2.5, 9.6] | 0.001 |
|  | Friday | **5.9** | [3, 8.9] | <0.001 | **8.4** | [4.9, 12] | <0.001 |
|  | Saturday | **-27.3** | [-30.3, -24.3] | <0.001 | **-54.1** | [-57.7, -50.4] | <0.001 |
|  | Sunday | **-40.9** | [-43.9, -37.9] | <0.001 | **-66.0** | [-69.6, -62.3] | <0.001 |
| **LPA** | Monday (cons) | 307.2 | [298.2, 316.3] | <0.001 | 284.9 | [275.7, 294.2] | <0.001 |
|  | Tuesday | -0.4 | [-2.6, 1.9] | 0.757 | -1.4 | [-3.9, 1.1] | 0.267 |
|  | Wednesday | **2.9** | [0.7, 5.2] | 0.010 | 0.1 | [-2.4, 2.5] | 0.955 |
|  | Thursday | **7.9** | [5.7, 10.2] | <0.001 | **2.9** | [0.5, 5.4] | 0.020 |
|  | Friday | **13.3** | [11.1, 15.6] | <0.001 | **10.7** | [8.2, 13.2] | <0.001 |
|  | Saturday | **9.2** | [6.9, 11.5] | <0.001 | **21.3** | [18.8, 23.9] | <0.001 |
|  | Sunday | **-4.1** | [-6.4, -1.8] | <0.001 | **6.8** | [4.3, 9.3] | <0.001 |
| **MVPA** | Monday (cons) | 22.5 | [20.5, 24.6] | <0.001 | 43.4 | [39.9, 46.8] | <0.001 |
|  | Tuesday | 0.2 | [-0.7, 1] | 0.701 | 1.3 | [-0.1, 2.7] | 0.076 |
|  | Wednesday | 0.7 | [-0.2, 1.5] | 0.139 | 0.6 | [-0.8, 2.1] | 0.388 |
|  | Thursday | **1.0** | [0.2, 1.9] | 0.019 | 1.2 | [-0.3, 2.6] | 0.108 |
|  | Friday | 0.1 | [-0.8, 0.9] | 0.887 | 0.6 | [-0.8, 2] | 0.410 |
|  | Saturday | **2.1** | [1.2, 3] | <0.001 | **10.8** | [9.3, 12.3] | <0.001 |
|  | Sunday | 0.4 | [-0.5, 1.2] | 0.433 | **8.8** | [7.3, 10.3] | <0.001 |
| **Season** | | | | | | | |
|  |  | **Female (n = 196)** | | | **Male (n = 148)** | | |
|  |  | Coeff | [95% CI] | p | Coeff | [95% CI] | p |
| **Sleep** | Summer (cons) | 498.7 | [490.5, 507] | <0.001 | 468.4 | [456.9, 479.9] | <0.001 |
|  | Autumn | **11.8** | [5.5, 18.2] | <0.001 | 8.4 | [-1, 17.8] | 0.081 |
|  | Winter | **16.2** | [9.9, 22.6] | <0.001 | **17.2** | [7.8, 26.7] | <0.001 |
|  | Spring | **7.7** | [1.3, 14.2] | 0.019 | 5.7 | [-3.8, 15.2] | 0.239 |
| **Sedentary** | Summer (cons) | 608.5 | [590.5, 626.4] | <0.001 | 615.2 | [598.1, 632.4] | <0.001 |
|  | Autumn | **-9.0** | [-16.9, -1] | 0.027 | **14.8** | [2.5, 27.1] | 0.018 |
|  | Winter | -4.5 | [-12.4, 3.5] | 0.268 | **18.9** | [6.6, 31.2] | 0.003 |
|  | Spring | **-14.0** | [-22, -5.9] | 0.001 | **14.1** | [1.7, 26.4] | 0.026 |
| **LPA** | Summer (cons) | 310.2 | [294.8, 325.6] | <0.001 | 312.7 | [300.5, 324.8] | <0.001 |
|  | Autumn | -2.4 | [-8.3, 3.5] | 0.430 | **-25.9** | [-34, -17.9] | <0.001 |
|  | Winter | **-10.3** | [-16.2, -4.4] | 0.001 | **-35.7** | [-43.7, -27.6] | <0.001 |
|  | Spring | 4.8 | [-1.1, 10.8] | 0.111 | **-23.2** | [-31.3, -15.1] | <0.001 |
| **MVPA** | Summer (cons) | 23.0 | [20.1, 26] | <0.001 | 45.7 | [40, 51.3] | <0.001 |
|  | Autumn | 0.2 | [-2.1, 2.4] | 0.876 | 1.9 | [-2.7, 6.6] | 0.422 |
|  | Winter | -1.0 | [-3.3, 1.2] | 0.365 | -1.3 | [-5.9, 3.4] | 0.595 |
|  | Spring | 2.2 | [-0.1, 4.5] | 0.057 | 3.2 | [-1.5, 7.9] | 0.188 |
| **Christmas - New Year** | | | | | | | |
|  |  | **Female (n = 200)** | | | **Male (n = 153)** | | |
|  |  | Coeff | [95% CI] | p | Coeff | [95% CI] | p |
| **Sleep** | pre (cons) | 488.4 | [480.6, 496.2] | <0.001 | 466.8 | [458, 475.7] | <0.001 |
|  | during | **28.0** | [21.9, 34.2] | <0.001 | **17.8** | [10.3, 25.3] | <0.001 |
|  | post | **24.3** | [17.8, 30.8] | <0.001 | **15.7** | [7.8, 23.6] | <0.001 |
| **Sedentary** | pre (cons) | 588.0 | [577.1, 598.9] | <0.001 | 610.0 | [596.6, 623.5] | <0.001 |
|  | during | 0.0 | [-7.3, 7.3] | 1.000 | **-11.3** | [-20.4, -2.1] | 0.016 |
|  | post | **16.9** | [9.1, 24.7] | <0.001 | 4.7 | [-4.9, 14.3] | 0.336 |
| **LPA** | pre (cons) | 344.3 | [330, 358.6] | <0.001 | 313.0 | [302.6, 323.4] | <0.001 |
|  | during | **-27.5** | [-33.1, -21.8] | <0.001 | -2.1 | [-8.6, 4.4] | 0.532 |
|  | post | **-40.5** | [-46.5, -34.4] | <0.001 | **-17.3** | [-24.1, -10.4] | <0.001 |
| **MVPA** | pre (cons) | 23.5 | [20.8, 26.1] | <0.001 | 51.4 | [47.1, 55.8] | <0.001 |
|  | during | 0.0 | [-2.2, 2.2] | 0.992 | **-4.5** | [-8.2, -0.8] | 0.018 |
|  | post | -0.5 | [-2.8, 1.8] | 0.691 | -3.5 | [-7.3, 0.4] | 0.079 |
| **DST start** | | | | | | | |
|  |  | **Female (n = 169)** | | | **Male (n = 122)** | | |
|  |  | Coeff | [95% CI] | p | Coeff | [95% CI] | p |
| **Sleep** | pre (cons) | 518.0 | [509.1, 526.9] | <0.001 | 484.9 | [474.1, 495.6] | <0.001 |
|  | post | 6.4 | [-1, 13.8] | 0.092 | 1.8 | [-7.1, 10.7] | 0.697 |
| **Sedentary** | pre (cons) | 595.7 | [570.4, 621] | <0.001 | 625.8 | [609.7, 641.9] | <0.001 |
|  | post | -6.7 | [-15.8, 2.4] | 0.151 | -1.1 | [-13, 10.9] | 0.863 |
| **LPA** | pre (cons) | 300.3 | [286.4, 314.1] | <0.001 | 284.6 | [272.1, 297.1] | <0.001 |
|  | post | -1.1 | [-7.7, 5.6] | 0.755 | -2.2 | [-9.9, 5.5] | 0.579 |
| **MVPA** | pre (cons) | 22.9 | [20.1, 25.7] | <0.001 | 48.9 | [43.9, 54] | <0.001 |
|  | post | -0.3 | [-2.8, 2.2] | 0.810 | -2.0 | [-6.5, 2.5] | 0.384 |
| **DST end** | | | | | | | |
|  |  | **Female (n = 188)** | | | **Male (n = 145)** | | |
|  |  | Coeff | [95% CI] | p | Coeff | [95% CI] | p |
| **Sleep** | pre (cons) | 505.5 | [498.2, 512.7] | <0.001 | 469.5 | [459.9, 479.2] | <0.001 |
|  | post | -6.6 | [-13.5, 0.3] | 0.061 | -7.7 | [-16.3, 0.9] | 0.078 |
| **Sedentary** | pre (cons) | 607.2 | [594.9, 619.5] | <0.001 | 634.6 | [619, 650.2] | <0.001 |
|  | post | 7.8 | [-0.4, 15.9] | 0.062 | 7.3 | [-3.7, 18.3] | 0.194 |
| **LPA** | pre (cons) | 305.8 | [295.1, 316.6] | <0.001 | 291.1 | [279.7, 302.5] | <0.001 |
|  | post | 0.1 | [-6, 6.2] | 0.973 | -1.3 | [-8.2, 5.5] | 0.704 |
| **MVPA** | pre (cons) | 22.2 | [19.6, 24.8] | <0.001 | 47.1 | [42.5, 51.7] | <0.001 |
|  | post | -0.2 | [-2.5, 2] | 0.831 | -0.3 | [-4.4, 3.8] | 0.903 |
| **School holidays** | | | | | | | |
|  |  | **Female (n = 209)** | | | **Male (n = 159)** | | |
|  |  | Coeff | [95% CI] | p | Coeff | [95% CI] | p |
| **Sleep** | school term (cons) | 501.5 | [495.9, 507.1] | <0.001 | 469.5 | [462.7, 476.2] | <0.001 |
|  | school holidays | **11.5** | [9.9, 13.0] | <0.001 | **11.3** | [9.5, 13.2] | <0.001 |
| **Sedentary** | school term (cons) | 600.7 | [583.4, 618.0] | <0.001 | 638.3 | [626.5, 650.1] | <0.001 |
|  | school holidays | **-6.4** | [-8.3, -4.6] | <0.001 | **-20.1** | [-22.4, -17.8] | <0.001 |
| **LPA** | school term (cons) | 317.6 | [200.1, 336.2] | <0.001 | 288.1 | [278.9, 297.3] | <0.001 |
|  | school holidays | **-4.4** | [-5.8, -3.0] | <0.001 | **6.1** | [4.5, 7.6] | <0.001 |
| **MVPA** | school term (cons) | 23.4 | [21.4, 25.4] | <0.001 | 45.9 | [42.5, 49.3] | <0.001 |
|  | school holidays | **-0.8** | [-1.3, -0.3] | 0.004 | **2.4** | [1.4, 3.3] | <0.001 |

Notes: Bold values indicate p < 0.05. CI = confidence interval, Coeff = coefficient, Cons = constant, LPA = light physical activity, MVPA = moderate-to-vigorous physical activity.


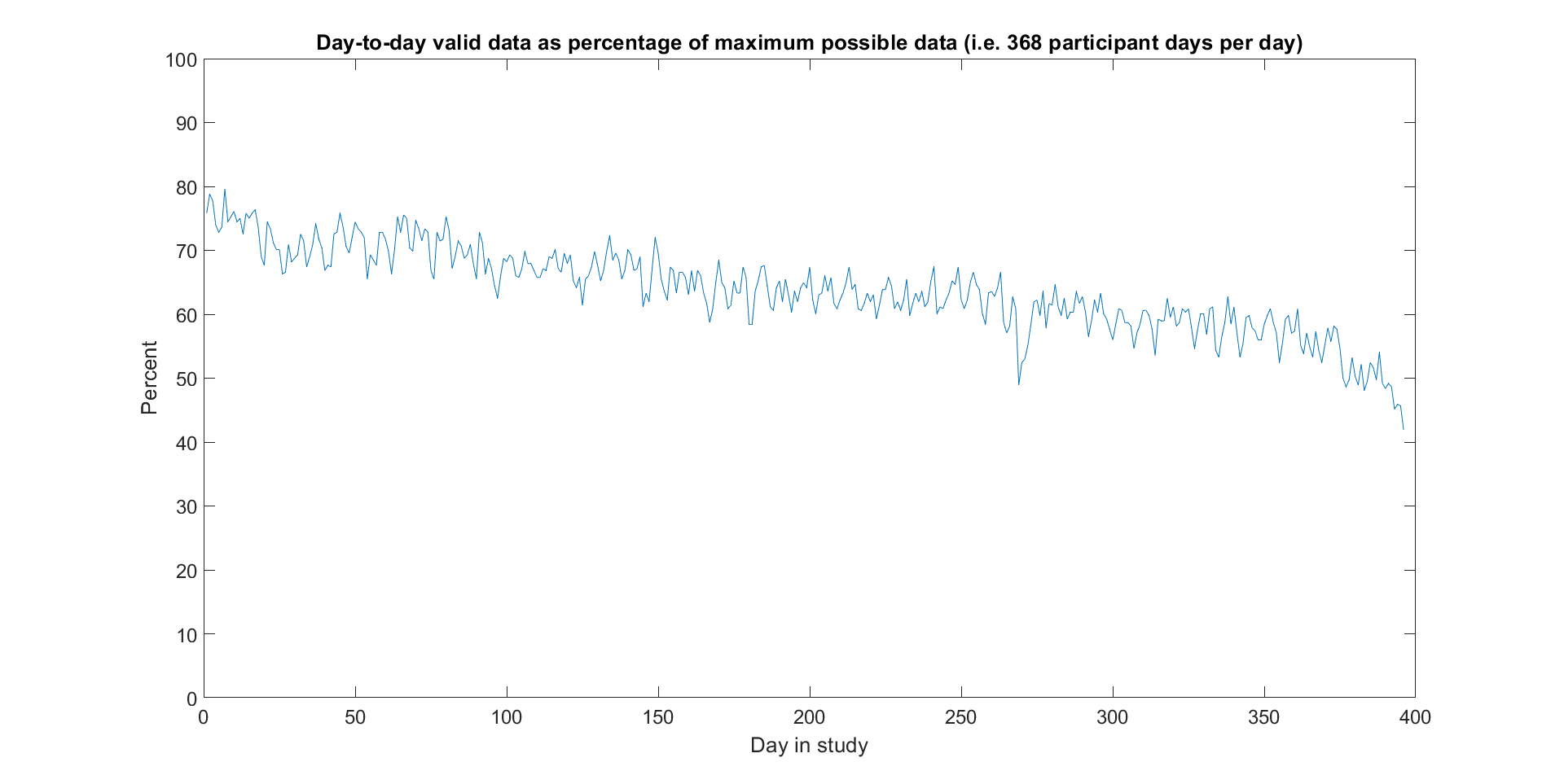


**Figure S1: Day-to-day valid data as percentage of maximum possible data**


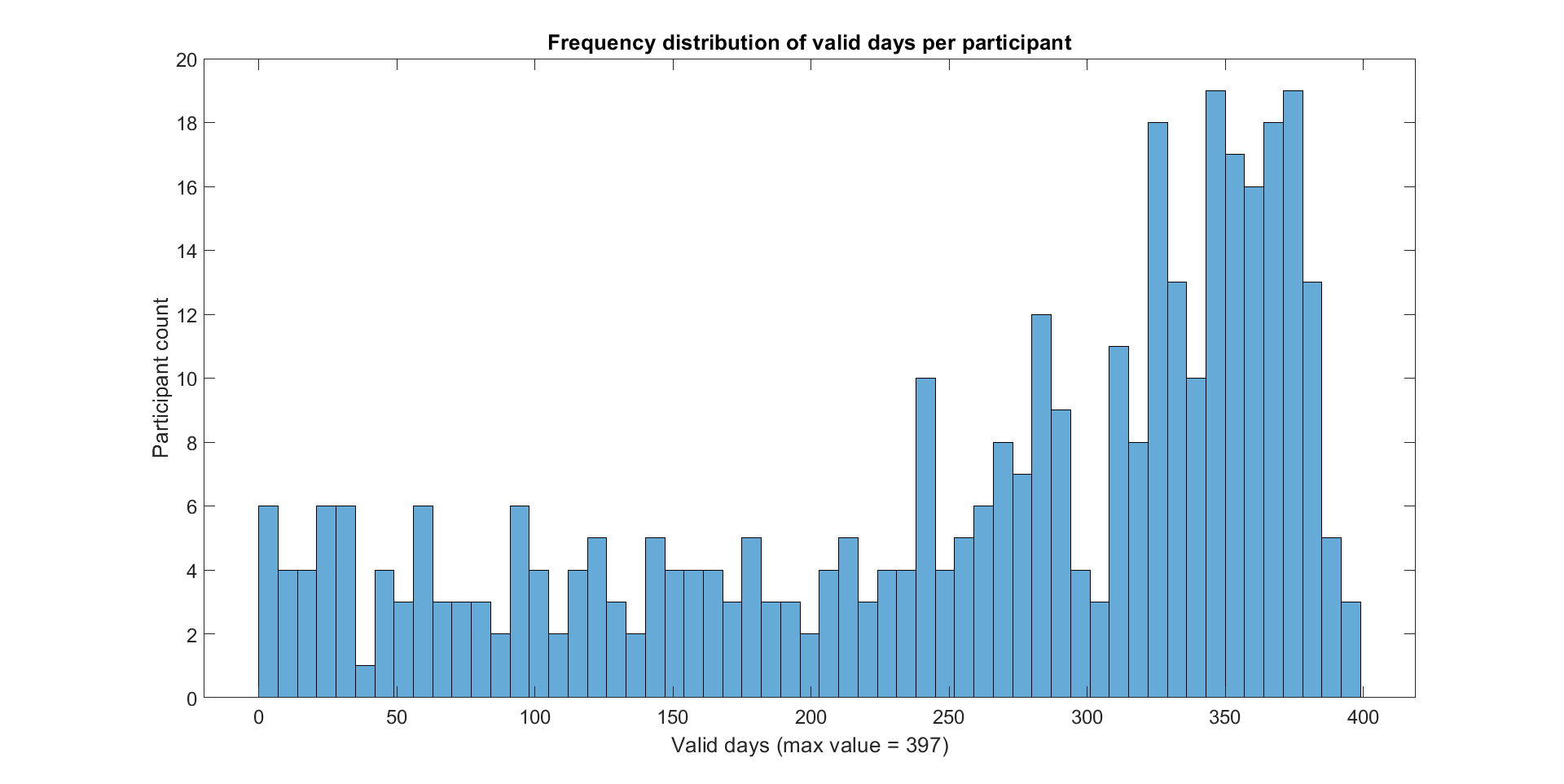


**Figure S2: Frequency distribution of valid days per participant**


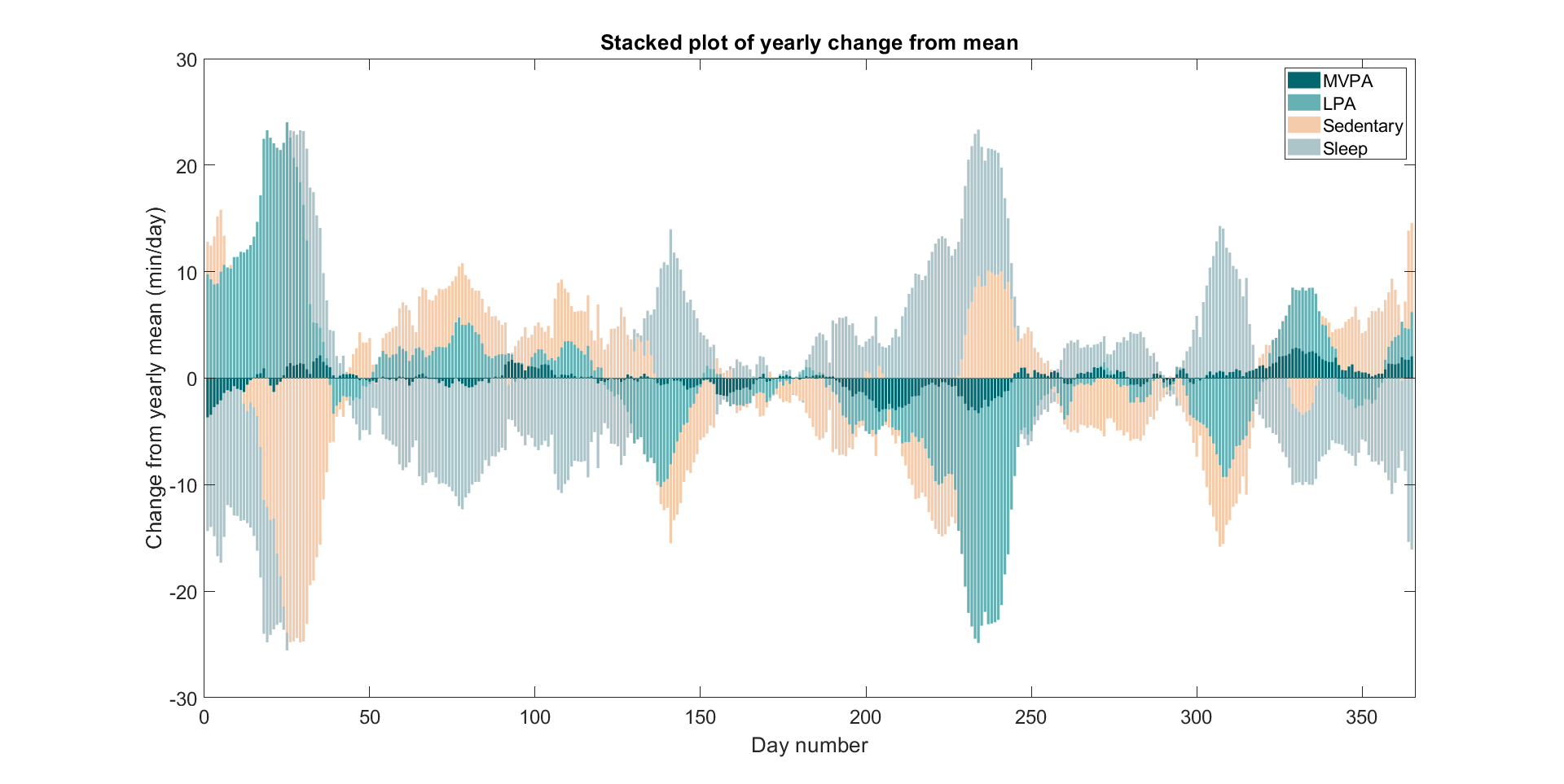


**Figure S3: Stacked plot of yearly change from mean**

Note: Each vertical column represents a single day with the highest and lowest points representing the sum of the positive and negative change behaviors respectively.
